# GPA-MDS: A Visualization Approach to Investigate Genetic Architecture among Phenotypes Using GWAS Results

**DOI:** 10.1155/2016/6589843

**Published:** 2016-10-27

**Authors:** Wei Wei, Paula S. Ramos, Kelly J. Hunt, Bethany J. Wolf, Gary Hardiman, Dongjun Chung

**Affiliations:** ^1^Department of Public Health Sciences, Medical University of South Carolina, Charleston, SC, USA; ^2^Department of Medicine, Medical University of South Carolina, Charleston, SC, USA; ^3^Ralph H. Johnson VA Medical Center, Charleston, SC, USA; ^4^Center for Genomic Medicine, Medical University of South Carolina, Charleston, SC, USA

## Abstract

Genome-wide association studies (GWAS) have identified tens of thousands of genetic variants associated with hundreds of phenotypes and diseases, which have provided clinical and medical benefits to patients with novel biomarkers and therapeutic targets. Recently, there has been accumulating evidence suggesting that different complex traits share a common risk basis, namely, pleiotropy. Previously, a statistical method, namely, GPA (Genetic analysis incorporating Pleiotropy and Annotation), was developed to improve identification of risk variants and to investigate pleiotropic structure through a joint analysis of multiple GWAS datasets. While GPA provides a statistically rigorous framework to evaluate pleiotropy between phenotypes, it is still not trivial to investigate genetic relationships among a large number of phenotypes using the GPA framework. In order to address this challenge, in this paper, we propose a novel approach, GPA-MDS, to visualize genetic relationships among phenotypes using the GPA algorithm and multidimensional scaling (MDS). This tool will help researchers to investigate common etiology among diseases, which can potentially lead to development of common treatments across diseases. We evaluate the proposed GPA-MDS framework using a simulation study and apply it to jointly analyze GWAS datasets examining 18 unique phenotypes, which helps reveal the shared genetic architecture of these phenotypes.

## 1. Introduction

Genome-wide association studies (GWAS) have been conducted to study the genetic basis of complex human traits. As of August 2015, more than 15,000 single nucleotide polymorphisms (SNPs) have been reported to be significantly associated with at least one complex trait (the NHGRI-EBI catalog of published GWAS [1], https://www.ebi.ac.uk/gwas/). “Pleiotropy,” that is, the sharing of genetic factors among complex traits, is well documented and a systematic analysis of the GWAS catalog of published GWAS showed that 17% of the reported genes are associated with multiple traits [2]. For example, genetic studies for five psychiatric disorders suggested a very strong genetic correlation between schizophrenia and bipolar disorder [3, 4]. Pleiotropy has also been demonstrated among several other types of traits, such as cancers [5].

In order to leverage pleiotropy between complex traits and effectively integrate multiple GWAS datasets, Chung et al. [6] developed a unified statistical framework, named GPA (Genetic analysis incorporating Pleiotropy and Annotation), which provides statistically rigorous and biologically interpretable inference tools for genetic studies. Application of GPA to five psychiatric disorder GWAS datasets from the Psychiatric Genomics Consortium [3, 4] showed that GPA can accurately identify pleiotropic structure among these diseases [6]. While the GPA framework provides a statistically rigorous framework to evaluate pleiotropy, it still remains limited to a small number of phenotypes and it is common to consider a joint analysis of only two phenotypes mainly due to computational efficiency and estimation stability and robustness. In practice, we are interested in jointly studying larger numbers of phenotypes; however it is not a trivial task to investigate and integrate results from multiple pairs of phenotypes.

In order to address this challenge, in this paper, we propose a novel visualization approach, GPA-MDS, to investigate genetic architecture with a joint analysis of multiple GWAS datasets using the GPA algorithm and the multidimensional scaling (MDS) approach. Specifically, the GPA algorithm allows for evaluation of pleiotropy between two phenotypes within a statistically rigorous framework. Then, the MDS approach effectively integrates these results for a large number of phenotypes and provides a two-dimensional map of genetic architecture. This paper is organized as follows. In Section 2, we review the GPA and MDS algorithms and propose the GPA-MDS approach. In Section 3, we evaluate the proposed method with a simulation study and apply it to a joint analysis of 18 GWAS datasets. Finally, in Section 4, we will discuss future research directions.

## 2. Methods: GPA-MDS Approach

**Figure 1:**
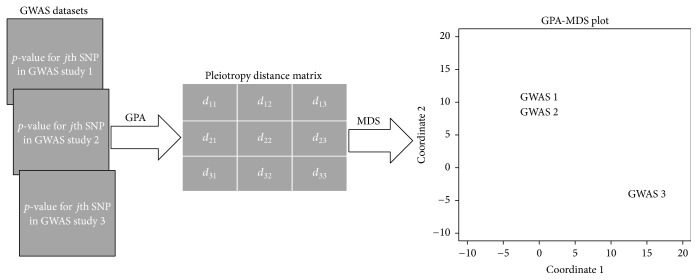
GPA-MDS framework. In this framework, by taking the association *p*-value for each SNP from each GWAS study as an input, we first calculate a distance matrix between phenotypes using the pleiotropy hypothesis testing procedures in the GPA framework. Then, we generate a plot depicting a global picture of genetic relationship among phenotypes by projecting phenotypes onto two-dimensional space using the multidimensional scaling (MDS) algorithm based on this distance matrix.

Figure 1 shows a diagram of overall workflow for the GPA-MDS framework. Here, by taking association *p*-value for each SNP from each GWAS dataset as an input, we first calculate a distance matrix between phenotypes using the GPA framework, as described in detail in Section 2.1. Then, we generate a plot of genetic relationship among phenotypes using the multidimensional scaling (MDS) algorithm, as illustrated in Section 2.2.

### 2.1. Statistical Inference of Pleiotropy Using the GPA Algorithm

In this section, we review the GPA framework [6] for the case of a joint analysis of two GWAS datasets. Although there is no limitation in the number of GWAS datasets that can be jointly analyzed in the GPA framework, a joint analysis of two GWAS datasets is often appropriate in the sense of the computational efficiency and estimation stability and robustness. Let *t* be the index for SNPs and let *k* be the index for GWAS datasets. Suppose that we have performed hypothesis testing of genome-wide SNPs for two GWAS and obtained their *p*-values. Specifically, for GWAS_1_, we have

(1) Null hypothesis for GWAS1:  H011,H021,…,H0t1,…,H0M1,p-value for GWAS1:  P11,P21,…,Pt1,…,PM1,

where *M* is the number of SNPs and *P*_*tk*_ denotes *p*-value of the *t*th SNP in the *k*th GWAS. Similarly, for GWAS_2_, we have

(2) Null hypothesis for GWAS2:  H012,H022,…,H0t2,…,H0M2,p-value for GWAS2:  P12,P22,…,Pt2,…,PM2.

Let us denote **P**_1_ = (*P*_11_, *P*_21_,…, *P*_*t*1_,…, *P*_*M*1_) and **P**_2_ = (*P*_12_, *P*_22_,…, *P*_*t*2_,…, *P*_*M*2_).

We introduce latent variables **Z**_*t*_ = [*Z*_*t*00_, *Z*_*t*10_, *Z*_*t*01_, *Z*_*t*11_] indicating the association between the *t*th SNP and the two phenotypes: *Z*_*t*00_ = 1 means that the *t*th SNP is not associated with any phenotypes, *Z*_*t*10_ = 1 means that it is only associated with the first one, *Z*_*t*01_ = 1 means that it is only associated with the second one, and *Z*_*t*11_ = 1 means that it is associated with both. We assume that *Z*_*t*00_, *Z*_*t*10_, *Z*_*t*01_, *Z*_*t*11_ ∈ {0,1} and *Z*_*t*00_ + *Z*_*t*10_ + *Z*_*t*01_ + *Z*_*t*11_ = 1 because a SNP can only be one of these states. Given these latent variables, we assume the following emission distributions:

(3) π00=Pr⁡Zt00=1:Pt1 ∣ Zt00=1~U0,1,  Pt2 ∣ Zt00=1~U0,1,π10=Pr⁡Zt10=1:Pt1 ∣ Zt10=1~Betaα1,1,  Pt2 ∣ Zt10=1~U0,1,π01=Pr⁡Zt01=1:Pt1 ∣ Zt01=1~U0,1,  Pt2 ∣ Zt01=1~Betaα2,1,π11=Pr⁡Zt11=1:Pt1 ∣ Zt11=1~Betaα1,1,  Pt2 ∣ Zt11=1~Betaα2,1,

where 0 < *α*_*k*_ < 1, *k* = 1,2. We put the constraint 0 < *α*_*k*_ < 1 to model that a smaller *p*-value is more likely than a larger *p*-value when it is from the nonnull group [7]. Parameters in the GPA model can be estimated using the Expectation-Maximization (EM) algorithm [8], which is remarkably computationally efficient because we have explicit solutions for all the parameters in the *M*-step. More details about the GPA model, the EM algorithm, and the estimation of standard errors can be found in [6].

Given the GPA model and its estimated parameters, we can determine association of *t*th SNP with phenotypes based on their local false discovery rate (FDR) [9]. Specifically, the local FDR for association of *t*th SNP with each of the first and second phenotypes is defined as

(4) fdr1Pt1,Pt2=Pr⁡Zt00+Zt01=0 ∣ Pt1,Pt2,fdr2Pt1,Pt2=Pr⁡Zt00+Zt10=0 ∣ Pt1,Pt2.

Similarly, the local FDR for association of *t*th SNP with both phenotypes is defined as

(5) $$\begin{array}{c} {fdr}_{12}\left(\;P_{t1},P_{t2}\;\right)=\mathrm{Pr}\left(\;Z_{t00}+Z_{t10}+Z_{t01}=0\;|\;P_{t1},P_{t2}\;\right). \end{array}$$

Then, we use the* direct posterior approach* [10] to control the global FDR. Specifically, we first sort SNPs by their local FDR from the smallest one to the largest one. Denote these sorted local FDR for *t*th SNP by *f*_*t*_. We increase the threshold for local FDR, *κ*, from zero to one until

(6) $$\begin{array}{c} {Fdr}_{\kappa }=\frac{\sum_{t=1}^{M}f_{t}1\left\{f_{t}\leq \kappa \right\}}{\sum_{t=1}^{M}1\left\{f_{t}\leq \kappa \right\}}\leq \tau , \end{array}$$

where *τ* is the predetermined bound of global FDR and 1{·} is an indicator function with value of one if the statement is true and of zero otherwise. Finally, we determine SNPs with corresponding *f*_*t*_ ≤ *κ* to be associated with the phenotype.

Now, consider testing pleiotropy between two phenotypes. When the genetic bases of the two phenotypes are independent of each other (i.e., no pleiotropy), then we expect that *π*_11_ = (*π*_10_ + *π*_11_)(*π*_01_ + *π*_11_). Therefore, the difference between *π*_11_ and (*π*_10_ + *π*_11_)(*π*_01_ + *π*_11_) can be used to characterize pleiotropy. Hence, testing pleiotropy can be formulated with the following hypothesis:

(7) $$\begin{array}{c} H_{0}\text{:}{\;\;\pi }_{11}=\pi_{1*}\pi_{*1},\;\;versus\;\;H_{1}\text{:}\;\;not\;\;H_{0}, \end{array}$$

where *π*_1*∗*_ = *π*_10_ + *π*_11_ and *π*_*∗*1_ = *π*_01_ + *π*_11_. The likelihood ratio test (LRT) statistic can be constructed as follows:

(8) $$\begin{array}{c} \lambda =\frac{\mathrm{Pr}\left(P_{1},P_{2};{\hat{\Theta }}_{0}\right)}{\mathrm{Pr}\left(P_{1},P_{2};\hat{\Theta }\right)}, \end{array}$$

where ${\hat{\Theta }}_{0}$ represents the parameter estimates obtained under the null hypothesis of pleiotropy test. The test statistic (−2log⁡*λ*) asymptotically follows *χ* ^2^-distribution with degree of freedom of one, under the null hypothesis. Fitting of the GPA model and hypothesis testing of pleiotropy were implemented as a part of the R package “GPA,” which is currently available in its GitHub page (http://dongjunchung.github.io/GPA/).

### 2.2. Visualization of Pleiotropic Structure Using Multidimensional Scaling

For the visualization of genetic relationships among phenotypes, we first run pleiotropy tests for all possible pairs of GWAS datasets and generate a matrix of their log_10_-transformed *p*-values, denoted as *s*_*ij*_. Then, we define a distance between *i*th and *j*th phenotypes as *d*_*ij*_ = *s*_*ij*_ − 2 min_*k*<*l*_{*s*_*kl*_}. Note that this definition of distance assigns a shorter distance to a pair of phenotypes with smaller pleiotropy test *p*-values, while it also allows avoiding negative distance values. Then, we feed this distance matrix to the MDS algorithm and project phenotypes onto the two-dimensional space. As a result, MDS essentially clusters phenotypes based on their genetic similarities; that is, phenotypes sharing fewer SNPs are located further apart on the two-dimensional space compared to phenotypes sharing more SNPs. Specifically, given a distance matrix *D* = (*d*_*ij*_), MDS seeks to find *x*_1_,…, *x*_*n*_ ∈ *R* ^*p*^ such that

(9) $$\begin{array}{c} d_{ij}\approx {\hat{d}}_{ij}={\left\|\;x_{i}-x_{j}\;\right\|}_{2} \end{array}$$

by minimizing the following objective function:

(10) $$\begin{array}{c} \frac{\sum_{i<j}{\left(d_{ij}-{\hat{d}}_{ij}\right)}^{2}}{\sum_{i<j}d_{ij}^{2}}. \end{array}$$

Here, we consider *p* = 2 to provide easily understandable visualization. We used the function cmdscale() in R with default settings to implement MDS.

## 3. Results

### 3.1. Simulation Study

**Figure 2:**
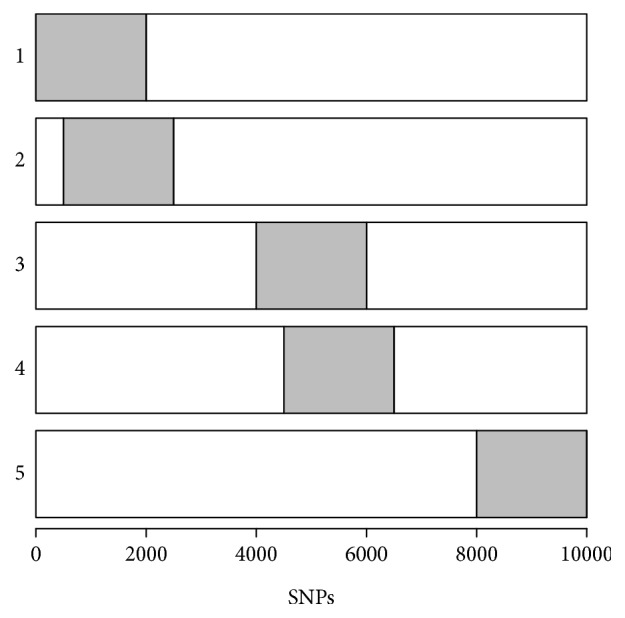
Design of simulation study. Each box indicates a GWAS study and *x*-axis represents the SNP index. The gray box within each box indicates risk SNPs. In this study, we considered five phenotypes and 20% of the SNPs were assumed to be risk SNPs for each phenotype. We further assumed that 75% of risk SNPs were shared between phenotypes 1 and 2 and also between phenotypes 3 and 4 to generate pleiotropic structure. Phenotype 5 did not share any risk SNPs with any other phenotypes as a negative control.

**Figure 3:**
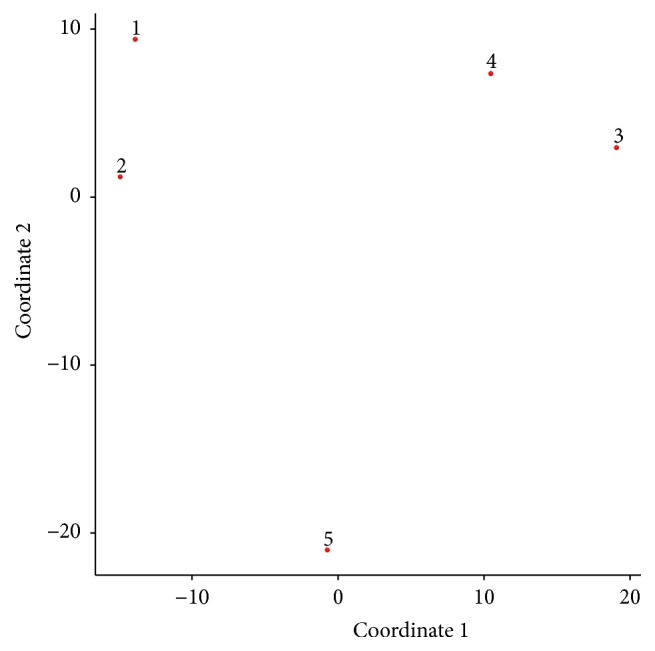
GPA-MDS plot for the simulation study.

We conducted a simulation study to evaluate the performance of GPA-MDS approach. Here, we assume that there are five GWAS datasets, each of which is profiled for a set of 10,000 SNPs common to all 5 datasets. Among these 10,000 SNPs, 2,000 SNPs (20%) were assumed to be risk SNPs for each phenotype. In order to generate pleiotropic structure, we set 75% of the risk SNPs to be shared between phenotypes 1 and 2 and also between phenotypes 3 and 4, while no risk SNPs are shared between phenotypes 1/2 and phenotypes 3/4. Phenotype 5 did not share any risk SNPs with any other phenotypes as a negative control (Figure 2). Finally, for each phenotype, we simulated *p*-values for nonrisk SNPs from a uniform distribution and *p*-values for risk SNPs from a Beta distribution with parameters 0.4 and 1.  Figure 3 shows the GPA-MDS plot for these five phenotypes. In this plot, phenotypes 1 and 2 are clustered and phenotypes 3 and 4 generate another cluster. Phenotype 5 is isolated and located away from these two phenotype clusters. This result shows that the proposed GPA-MDS approach can provide easily interpretable visualization revealing the pleiotropic architecture among phenotypes.

### 3.2. Real Data Analysis

We applied the proposed GPA-MDS approach to the GWAS datasets of 18 phenotypes, using summary statistics, which are publicly available from consortium websites. Specifically, we considered (1) attention deficit/hyperactivity disorder (ADHD), autism spectrum disorder (ASD), bipolar disorder (BPD), major depressive disorder (MDD), and schizophrenia (SCZ) from the Psychiatric Genomics Consortium (http://www.med.unc.edu/pgc); (2) Crohn's disease (CD) and ulcerative colitis (UC) from the International Inflammatory Bowel Disease Genetics Consortium (https://www.ibdgenetics.org/); (3) rheumatoid arthritis (RA) (https://www.broadinstitute.org/ftp/pub/rheumatoid_arthritis/Stahl_etal_2010NG/); (4) high-density lipoprotein (HDL), low-density lipoprotein (HDL), triglycerides (TG), and total cholesterol (TC) from the Global Lipids Consortium (http://csg.sph.umich.edu//abecasis/public/lipids2010/); (5) type 2 diabetes (T2D) from the DIAbetes Genetics Replication And Meta-analysis Consortium (http://diagram-consortium.org/); (6) coronary artery disease (CAD) from the CARDIoGRAM Consortium (http://www.cardiogramplusc4d.org/data-downloads/); (7) systolic blood pressure (SBP) and diastolic blood pressure (DBP) from the International Consortium for Blood Pressure (http://www.georgehretlab.org/icbp_088023401234-9812599.html); and (8) fasting glucose (FG) and log of fasting insulin (LFI) from the MAGIC Consortium. We used the intersection of SNPs among these datasets, which consists of 228,944 SNPs.

**Figure 4:**
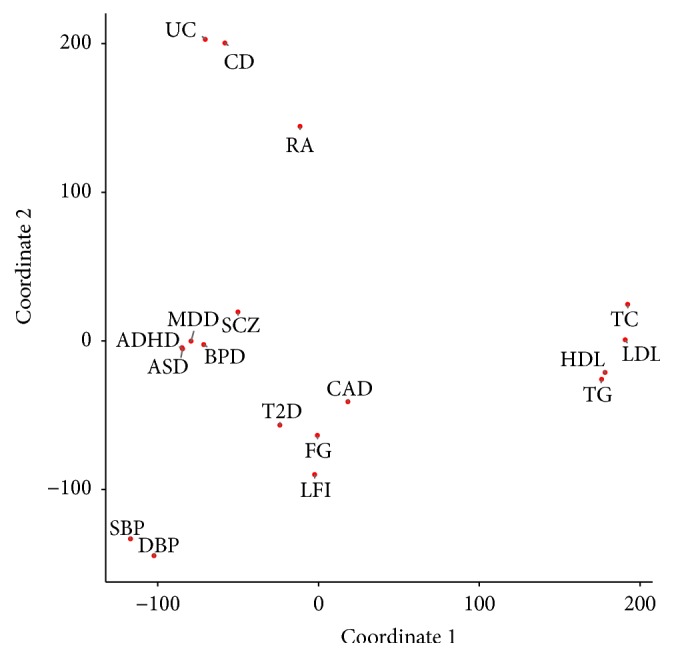
GPA-MDS plot for a joint analysis of genetic studies for 18 phenotypes.

Figure 4 shows the GPA-MDS plot for the 18 phenotypes. We can see that clinically related phenotypes are tightly clustered in this plot. For example, all the neuropsychiatric disorders (ADHD, ASD, BPD, MDD, and SCZ) generate a cluster, inflammatory bowel diseases (UC and CD) make a cluster, lipid-related phenotypes (HDL, LDL, TC, and TG) cluster together, blood pressure phenotypes (SBP and DBP) cluster, and so on. Moreover, RA is also located relatively close to UC and CD, which is consistent with the literature as RA, UC, and CD are all autoimmune diseases [11]. The cluster containing both T2D and CAD in this plot is also well supported by prior studies, which suggest the pleiotropy between T2D and CAD [12–14]. These results show the potential of the proposed GPA-MDS approach for the investigation of pleiotropic architecture, which can be used to promote understanding of common etiology and development of joint treatment of diseases.

**Figure 5:**
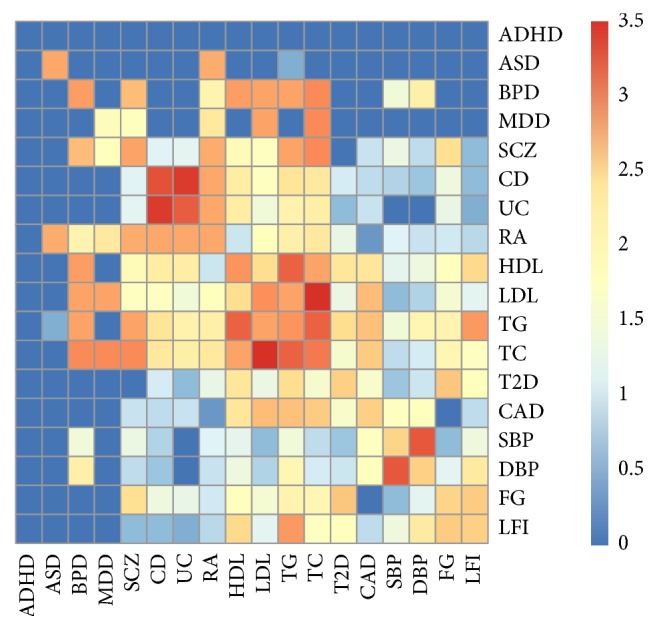
Heat map of shared risk SNPs for 18 phenotypes. Here, “risk SNPs” were determined using the GPA algorithm by controlling the global FDR at 0.1. Then, values were log_10_-transformed for the visualization purpose.

In order to further understand the phenotype mapping provided by GPA-MDS, we checked the number of risk SNPs shared among phenotypes (Figure 5). Here, “risk SNPs” were determined using the GPA algorithm by controlling the global FDR at 0.1. We can see that some of the phenotypes that are closely located in the GPA-MDS plot actually share more risk SNPs, as in the case of CD-UC, HDL-LDL-TG-TC, and SBP-DBP. However, it might look like that Figure 5 seems to contradict the GPA-MDS plot for other phenotypes. For example, although ADHD and ASD are located really close to each other in the GPA-MDS plot, it seems that only a few risk SNPs are shared between these two phenotypes. Such “discrepancy” happens because GPA evaluates pleiotropy by checking whether the number of shared risk SNPs (*π*_11_) is significantly higher than what is expected by chance ((*π*_10_ + *π*_11_)(*π*_01_ + *π*_11_)), not simply based on the counts of shared risk SNPs (*π*_11_).

**Figure 6:**
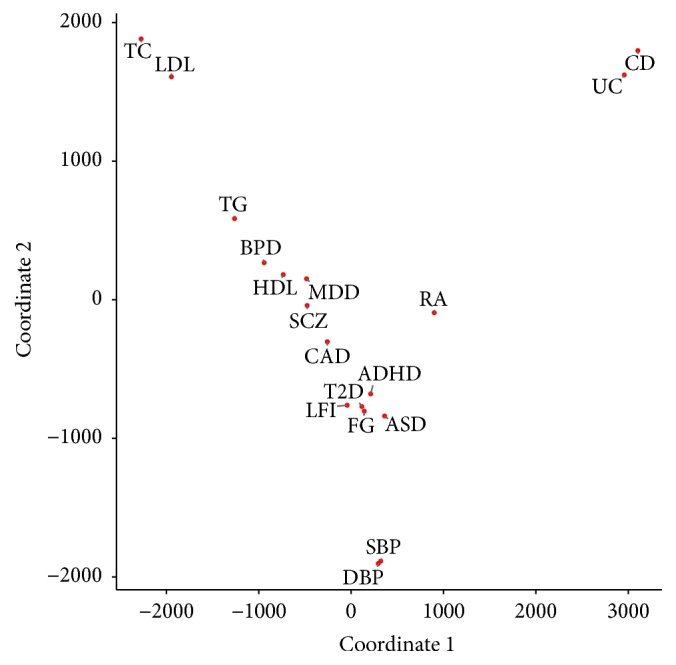
MDS plot for a joint analysis of genetic studies for 18 phenotypes, based on the number of risk SNPs shared between two phenotypes. Here, “risk SNPs” were determined using the GPA algorithm by controlling the global FDR at 0.1.

In order to confirm this explanation and further evaluate the utility of employing the GPA algorithm, we generated a MDS plot of phenotypes, where the distance between two phenotypes was determined solely by the number of shared risk SNPs (Figure 6). Specifically, we generated the MDS plot by defining the distance between *i*th and *j*th phenotypes as *d*_*ij*_′ = 2 max_*k*<*l*_{*n*_*kl*_} − *n*_*ij*_, where *n*_*ij*_ is the log_10_-transformed risk SNPs shared between *i*th and *j*th phenotypes. In this mapping of phenotypes, clinically related phenotypes failed to cluster together but instead multiple phenotype groups are mixed together. Furthermore, we can see that the mapping is essentially driven by a few pairs of phenotypes which share large numbers of genotypes, such as CD-UC, TC-LDL, and SBP-DBP. Hence, we can conclude that it was actually critical to utilize the GPA algorithm to evaluate pleiotropy in the first step of our visualization framework because it provides biologically more meaningful visualization of genetic relationship among phenotypes.

## 4. Conclusion

In this paper, we proposed a novel visualization approach for genetic architecture among phenotypes, namely, GPA-MDS, using the GPA algorithm and the multidimensional scaling (MDS) approach. While the GPA framework provides a rigorous evaluation of pleiotropy between a pair of phenotypes, the MDS approach extends this investigation to larger number of phenotypes in a computationally efficient way. The application of GPA-MDS to the genetic studies of 18 phenotypes revealed patterns of shared genetic architecture among phenotypes, underscoring the potential of the proposed method to investigate genetic sharing among complex traits. We note that when the proposed GPA-MDS framework is used, it is critical to confirm that its assumptions hold well for the input GWAS dataset. Specifically, because GPA-MDS uses GPA as its first step, users need to confirm that GWAS association *p*-values provided to GPA-MDS satisfy the GPA assumptions, for example, uniformity of null *p*-values. For example, if population stratification and cryptic relatedness have not been accounted for in previous GWAS studies, *p*-values of null SNPs may not follow the assumed uniform distribution and can result in biased MDS visualization results. Hence, these confounding effects should be checked carefully and addressed before applying the GPA-MDS framework to these GWAS association *p*-values. We recommend readers to check [6] for deeper discussion of the GPA assumptions.

Currently, we are working on the following directions that can further improve the GPA-MDS framework. First, in this paper, we used a definition of distance based on the logarithm transformation of *p*-values. While this approach is intuitive and works well in practice, other choices of distance measures might change visualization results. Hence, it is of great interest to investigate other choices of distance measures and their impacts on visualization results. Second, while the proposed GPA-MDS approach promotes global understanding of genetic architecture, it is still laborious to pinpoint risk SNPs leading to phenotype clusters when we investigate a large number of phenotypes. Hence, it would be desirable to automate the procedure to identify overlapping risk SNPs. We expect that GPA-MDS will be a useful method in elucidating the pleiotropic architecture of complex traits, which can contribute to a better understanding of shared genetic mechanisms and the development of improved diagnosis and therapeutics.
